# Multiscale Simplicial Complex Entropy Analysis of Heartbeat Dynamics

**DOI:** 10.3390/e27050467

**Published:** 2025-04-25

**Authors:** Alvaro Zabaleta-Ortega, Carlos Carrizales-Velazquez, Bibiana Obregón-Quintana, Lev Guzmán-Vargas

**Affiliations:** 1 Laboratorio de Sistemas Complejos, Unidad Interdisciplinaria en Ingeniería y Tecnologías Avanzadas, Instituto Politécnico Nacional, Av. IPN No. 2580, L. Ticomán, Ciudad de México 07340, Mexico; 2 Facultad de Ciencias, Universidad Nacional Autónoma de México, Ciudad de México 04510, Mexico

**Keywords:** multiscale entropy, heartbeat dynamics, simplicial complex

## Abstract

The present study proposes a multiscale analysis of the simplicial complex approximate entropy (MS-SCAE) applied to cardiac interbeat series. The MS-SCAE method is based on quantifying the changes in the simplicial complex associated with the time series when a coarse-grained procedure is performed. Our findings are consistent with those of previously reported studies, which indicate that the complexity of healthy interbeat dynamics remains relatively stable over different scales. However, these dynamics undergo changes in the presence of certain cardiac pathologies, such as congestive heart failure and atrial fibrillation. The method we present here allows for effective differentiation between different dynamics and is robust in its ability to characterize both real and simulated sequences. This makes it a suitable candidate for application to a variety of complex signals.

## 1. Introduction

The evaluation of the complexity present in physiological signal fluctuations offers the opportunity to identify properties of the functioning of living systems [1]. Among the most commonly used methodologies are those from information theory or entropy-based methodologies. Entropy is defined as the average uncertainty of a random variable. In order to estimate the average rate of generation of information provided by a new observation given *n* previous observations, it is necessary to resort to definitions such as the Kolmogorov–Sinai entropy, which is useful for characterizing dynamical systems [2]. The underlying premise of these methodologies is to quantify the complexity of a given system by measuring the degree of irregularity of a sequence of observations. However, the estimation of these measures is challenging because they can be underestimated when the number of observations is finite. In 1991, Pincus [3] introduced approximate entropy (ApEn) as a measure of complexity in some low-dimensional chaotic and stochastic systems. Subsequently, Richman and Moorman [4] adapted and refined this strategy by neglecting the self-matches of the model vector, thereby introducing the concept of sample entropy (SampEn). These complexity quantifiers and their adaptations for the case of time-evolving systems have been especially useful in the analysis of complex physiological time series [5,6,7,8,9,10,11,12,13,14,15,16].

In this context, the irregularity of the interbeat interval (also known as the RR interval, which represents the duration of a ventricular cardiac cycle, measured between two successive R-wave peaks) has attracted attention because it shows fluctuations with long-term correlations in healthy conditions. Furthermore, the RR interval reflects the dynamic interactions between the autonomic nervous system and cardiac function, whose properties degrade with disease or aging [17,18,19,20,21,22]. The quantification of complexity in this variable has been the subject of multiple studies ranging from frequency, fractal and multifractal analysis to entropy production [23,24,25,26,27,28,29,30]. In 2002, Costa et al. [31] introduced the multiscale entropy analysis (MSE) to quantify the changes in the degree of unpredictability of the heartbeat time series across multiple scales [31]. MSE has been extensively utilized in various research domains, including geo-electric activity time series [32], financial time series [33], image texture evaluation [34], and mechanical vibrations [35], among others.

Costa et al. provided evidence that the complexity of beat-to-beat time series under a coarse-graining procedure undergoes significant changes in the presence of disease [6]. In contrast, under healthy conditions and under equivalent levels of coarse-graining, complexity remains relatively constant across multiple scales [6,36]. For time series with a length of *N*, the length of coarse-grained time series diminishes to N/τ when the scale factor is designated as τ. This observation indicates that coarse-grained time series undergo a substantial reduction in length as the scale factor increases [32]. In response to this phenomenon, a number of modified multiscale entropy methods have been proposed. One such approach, composite multiscale entropy (CMSE), was proposed by Wu et al. [37] to enhance the stability of entropy values at large scales. Valencia et al. [38] introduced the refined multiscale entropy (RMSE) to address the MSE bias that arises from the removal of fast time scales during the averaging procedure. Subsequently, Liu et al. [39] developed generalized refined multiscale entropy (RGMSE) in 2018, a method that considers higher moments in the coarse-grained process instead of the first moment (mean values). Subsequent studies have proposed the utilization of alternative entropy definitions. For instance, Li et al. [28] proposed a novel distribution entropy algorithm (DisEn), while Lee et al. [40] introduced the multiscale distribution entropy (MDE) in 2018. The MDE is calculated by the DisEn values of coarse-grained time series obtained by the rolling averaging process. In a similar vein, the modified Renyi multiscale distribution entropy has recently been introduced [41], operating through a rolling average technique to calculate the Renyi distribution entropy for coarse-grained time series.

Although MSE and its variants (see [37,42,43]) have shown important differences in “entropy spectra” when comparing groups of healthy and diseased subjects, it is important to identify when these differences are truly due to the underlying dynamics whose properties are preserved in the coarse-graining process. Here, we approach this problem from a perspective based on the study of the topological features of complex signals such as the cardiac interbeat interval.

We have recently introduced an alternative version of entropy to measure the irregularity of time series based on constructing simplicial complexes from time series called simplicial complex approximate entropy (SCAE), defined as the conditional probability that two elements of the simplicial complex (of the same dimension *k*) that are “close” will remain “close” to a third element, forming a simplex of higher dimension (k+1) [44]. SCAE is rooted in persistent homology [45,46,47,48,49], a topological data analysis tool [50,51,52] that uses the “proximity” notion to extracting geometrical data properties by making multi-resolution scale filtrations of a dataset arranged as a point cloud. In simple words, given a data set, it is arranged as a *d*-dimensional point cloud, and simplexes are constructed by connecting any points within a given distance ϵ. The filtering corresponds to varying the distance parameter ϵ over $0\leq \epsilon <\epsilon_{max}$. The collection of simplexes resulting from such a filtration forms a simplicial complex containing the geometric information of the dataset, typically condensed in a persistence diagram or a bar code containing the persistences of the homology groups formed in the filtration. The main difference between the SCAE and ApEn (or SampEn) involved in the MSE analysis lies in the fact that in the case of the SCAE, it considers the matches that exist between patterns of the same length when the simplex dimension is increased by one, but the length of the patterns remains constant (see refs. [3,4,5,44,53] for details). From a general point of view, it is interesting to evaluate the complexity of a signal on different scales by observing the behavior of the conformation of local structures (simplexes) as a function of their dimension, which allows to distinguish dynamics with different levels of irregularity in a clearer way. In this paper, we address this question in order to contribute to the discussion of the complexity of time series in a multiscale approach. The structure of the paper is as follows. In Section 2, we present a brief description of the SCAE method and the coarse-grained procedure. The results on real and simulated data are presented in Section 3. Finally, the discussion and conclusions are presented in Section 4.

## 2. Methods and Database

We briefly describe the main steps to compute the SCAE [44]. Given a time series {x(i)}={x(1),x(2),⋯,x(N)} with *N* values, *d*-dimensional vectors (patterns) are considered to construct the point cloud $PC_{d}=\left\{\left(x_{j},x_{j+1},\ldots ,x_{j+d-1}\right)\right\}$ with 1≤j≤N−d+1 data points. Next, the Vietoris–Rips (VR) simplicial complex is constructed by linking any pair of points ($x_{p},x_{q}$) under the condition $\left|\right|x_{p},x_{q}{\left|\right|}_{2}\leq \epsilon$ (we set ϵ as a factor of the standard deviation of the time series), forming the simplexes $\sigma_{k}$ of dimension *k* (see [54,55,56,57] for details about the Vietoris–Rips construction). In the metrical space, a $\sigma_{0}$ is a point, a $\sigma_{1}$ is a line segment (an edge), a $\sigma_{2}$ is a triangle, a $\sigma_{3}$ is a tetrahedron, and so on for higher dimensional polyhedral (detailed theoretical information about the simplicial complex can be seen in refs. [45,46,47,48,49]). Next, we count the number of simplexes $\rho_{k}$ of dimension *k* in the simplicial complex and compute the normalized quantity $S_{k}=\rho_{k}/\rho_{k}^{m}$, with respect to the maximum number of simplexes $\rho_{k}^{m}$ of dimension *k* that can be formed in the simplicial complex. Similarly, we compute $S_{k+1}=\rho_{k+1}/\rho_{k+1}^{m}$ for simplices of dimension k+1. The maximum number of simplices $\rho_{k}^{m}$ and $\rho_{k+1}^{m}$ in the simplicial complex corresponds to the combinations ρkm=N−d+1k+1 and ρk+1m=N−d+1k+2 for dimensions *k* and k+1, respectively. Finally, we define SCAE_*k*_ = $-\ln(S_{k+1}/S_{k})$ [44]. For k=0, SCAE_0_ is the conditional probability that a given point will form an edge with a neighboring point. Similarly, for k=1, SCAE_1_ is the conditional probability that two points that are near each other and form an edge will also form part of a triangle with a third point that is also near. A lower value of either SCAE_0_ or SCAE_1_ is indicative of a more regular behavior of the sequence, whereas high values are assigned to more irregular series.

**Figure 1:**
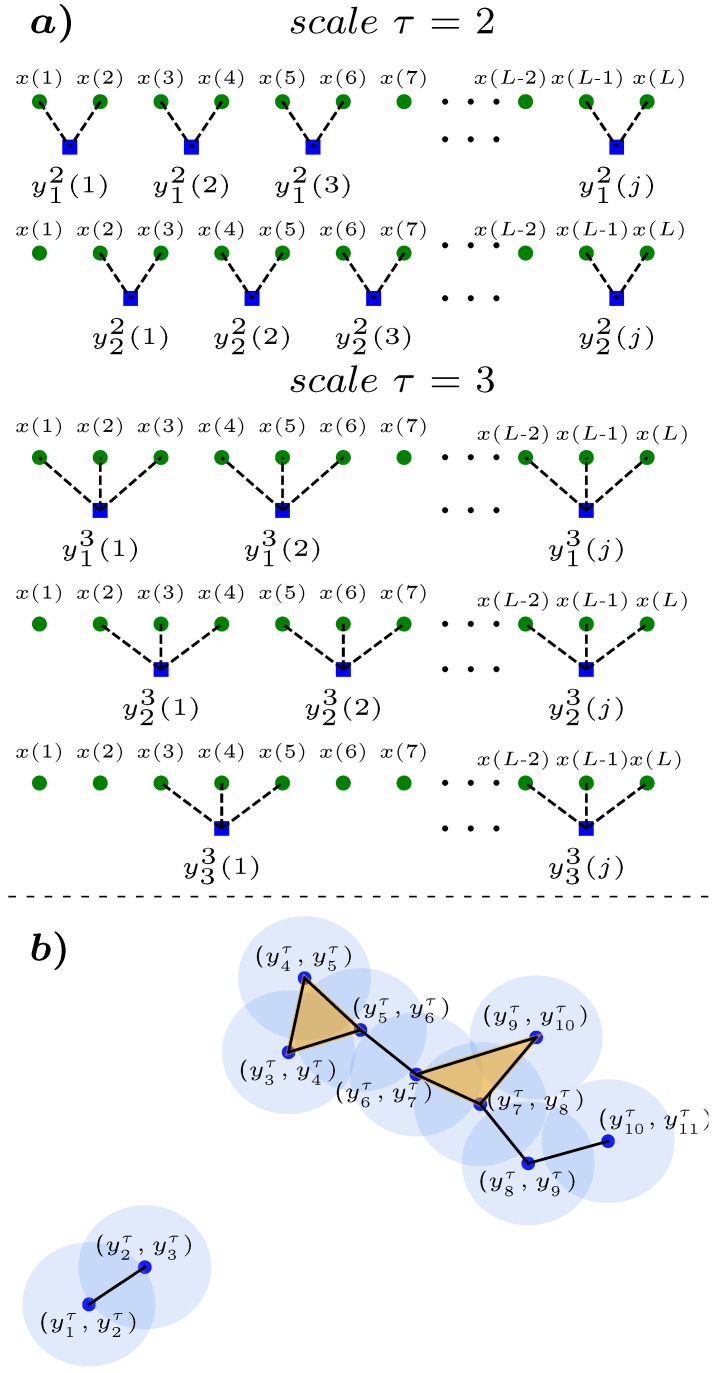
MS-SCAE procedure illustration. For a time series {x(t)}, at each scale τ we set the corresponding coarse-grained time series $y_{r}^{\tau }\left(j\right)$ (see main text). For example, at scale τ=2, we have two subseries, $y_{1}^{2}\left(j\right)=\left[x\left(j\right)+x\left(j+1\right)\right]/2$, $y_{2}^{2}\left(j\right)=\left[x\left(j+1\right)+x\left(j+2\right)\right]/2$, at scale τ=3, we have three subseries, $y_{1}^{3}\left(j\right)=\left[x\left(j\right)+x\left(j+1\right)+x\left(j+2\right)\right]/3$, $y_{2}^{3}\left(j\right)=\left[x\left(j+1\right)+x\left(j+2\right)+x\left(j+3\right)\right]/3$, $y_{3}^{3}\left(j\right)=\left[x\left(j+2\right)+x\left(j+3\right)+x\left(j+4\right)\right]/3$, as depicted in top and bottom of (**a**), respectively, where L=[(N//τ)∗τ]−r+1, (where N//τ represents the modulus of *N* regarding τ). Next, using the point cloud $PC_{d=2}=\left\{\left(y^{\tau }\left(j\right),y^{\tau }\left(j+1\right)\right)\right\}$ coming from the coarse-grained time series $\left\{y^{\tau }\left(j\right)\right\}$ at the given scale τ, we construct the Vietoris–Rips (VR) simplicial complex as exemplified in (**b**) for an arbitrary sub-series. In this illustrative example, the value of ϵ is denoted by the border of the shaded area in light blue. The VR simplicial complex consists of P=10 points and the number of simplexes are: $\rho_{0}=10$, $\rho_{1}=10$ and $\rho_{2}=2$. The maximum number of simplexes for dimension k=0 is ρ0m=101=10, for k=1 is ρ1m=102=45 and for k=2 is ρ2m=103=120. Their respective quotients are: $S_{0}=\rho_{0}/\rho_{0}^{m}=10/10=1$, $S_{1}=\rho_{1}/\rho_{1}^{m}=10/45$, and $S_{2}=\rho_{2}/\rho_{2}^{m}=2/120$. Finally, we calculate their corresponding entropy given by SCAE_0_ = $-\ln\left[S_{1}/S_{0}\right]=-\ln\left[10/45\right]\approx 0.654$, and SCAE_1_ = $-\ln\left[S_{2}/S_{1}\right]=-\ln\left[\left(2/120\right)/\left(10/45\right)\right]\approx 1.123$.

The multiscale procedure consists of two main steps: (i) the application of a coarse-graining procedure, and (ii) the quantification of the irregularity (SCAE) of the coarse-grained time series. Regarding (i), given a one-dimensional time series {x(t)}=x(1),x(2),⋯,x(N) consisting of *N* observations, we construct *r* coarse-grained time subseries (1≤r≤τ) by computing the arithmetic mean over w=(N/τ)−r+1 windows of size τ [37]. The *j*-th coarse-grained point of the *r*-th subseries at the given scale τ is calculated according to the expression $y_{r}^{\tau }\left(j\right)=\frac{1}{\tau }\sum_{l=(j-1)\tau +r}^{j\tau +r-1}x\left(l\right)$. Thus, the coarse-grained time series at scale τ is the collection $\left\{y_{r}^{\tau }\left(j\right)\right\}$ for 1≤j≤w and 1≤r≤τ (this procedure is illustrated in Figure 1a). It is important to emphasize that the coarse graining procedure functions as a low pass filter, with the principal role being played by the lower frequencies of the time series under consideration [38]. Finally and with respect to (ii), we compute the simplicial complex approximate entropy 〈SCAE_*k*_$\left(\tau \right)\rangle =-\frac{1}{\tau }\sum_{r=1}^{\tau }\ln\left(S_{k+1}^{\tau ,r}/S_{k}^{\tau ,r}\right)$, where $S_{k}^{\tau ,r}$ and $S_{k+1}^{\tau ,r}$ are the probabilities computed over the *r*-th coarse-grained time subseries $y_{r}^{\tau }\left(j\right)$ at scale τ (see Figure 1b).

The MS-SCAE method is applied to heartbeat interval (RR) time series derived from electrocardiographic (ECG) recordings of a group of healthy subjects, patients with congestive heart failure (CHF), and patients with atrial fibrillation [58,59]. CHF is a life-threatening condition in which the heart is unable to pump blood efficiently enough to meet the body’s need for oxygen and nutrients and atrial fibrillation (AF) is a type of arrhythmia characterized by irregular and often rapid heartbeats. The heartbeat interval time series analyzed are: 16 healthy subjects (mean age 32.6 years), 12 patients with congestive heart failure CHF (mean age 54.4 years) and 10 patients with atrial fibrillation [58]. For the atrial fibrilation group, the recordings were filtered to exclude artifacts, premature ventricular complexes, and undetected beats. This database is part of an extended set of records used in previous studies [6,24,60,61,62]. In our study, RR interval sequences of approximately $2\times 10^{4}$ beats, corresponding to approximately four daily hours of ECG recordings, were considered. For efficient data processing, we calculated SCAE_0_ and SCAE_1_ for two non-overlapping segments of $10^{4}$ values for each subject and then considered the average of them. In our calculations, we set ϵ=0.1, d=2, and k=0 (or 1).

## 3. Results

**Figure 2:**
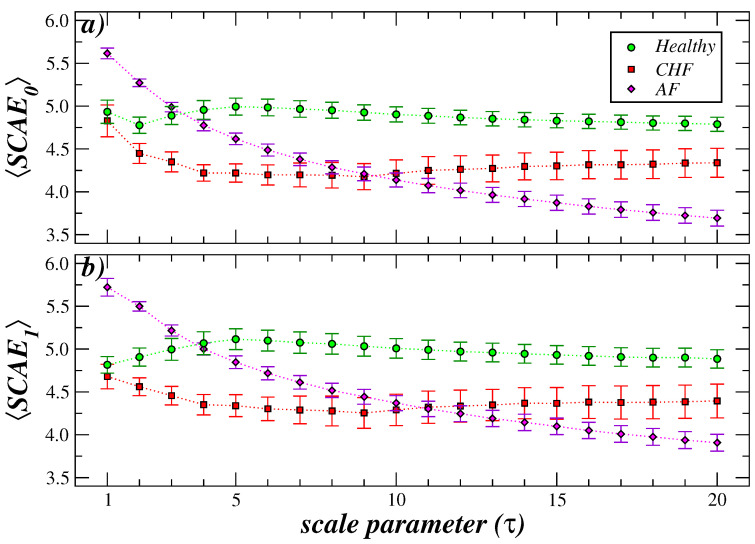
MS-SCAE analysis of interbeat cardiac series from healthy subjects, patients with congestive heart failure, and patients with atrial fibrillation. We show the results for SCAE_0_ (**a**) and SCAE_1_ (**b**) for the three groups. Symbol represents the mean values of entropy of each group and bars represent the standard error ($SD/\sqrt{n}$, with *n* the number of subjects). For both entropies, the three groups behave differently as τ grows. While the entropy values of the healthy group increase slightly for short scales and then decrease moderately, the heart failure group decreases slightly and then increases its value, always below the values of the healthy group. In contrast, the patients with atrial fibrillation show a high value for small scales and a rapid decrease as τ increases, so that for larger scales (τ≥12), the entropy values are lower than the other two groups.

Initially, the impact of coarse-graining the cardiac interbeat sequences from the three groups in our study was examined. Figure 2 shows the results of the behavior of SCAE_0_ and SCAE_1_ for healthy subjects, patients with congestive heart failure (CHF) and subjects with atrial fibrillation (AF) as a function of τ. We highlight the fact that at τ=1, the three groups are separated, with the entropy of the atrial fibrillation group being greater than that of the healthy and CHF groups. As τ increases, the three groups behave differently. In the healthy group, SCAE_0_ shows a slight decrease at τ=2 and then increases to a plateau, while SCAE_1_ shows a slight increase and then gradually decreases. In CHF patients, both entropy measures show a small decrease and then increase. The entropy of the AF group steadily decreases to values that are lower than those estimated for the CHF group and for healthy subjects.

**Figure 3:**
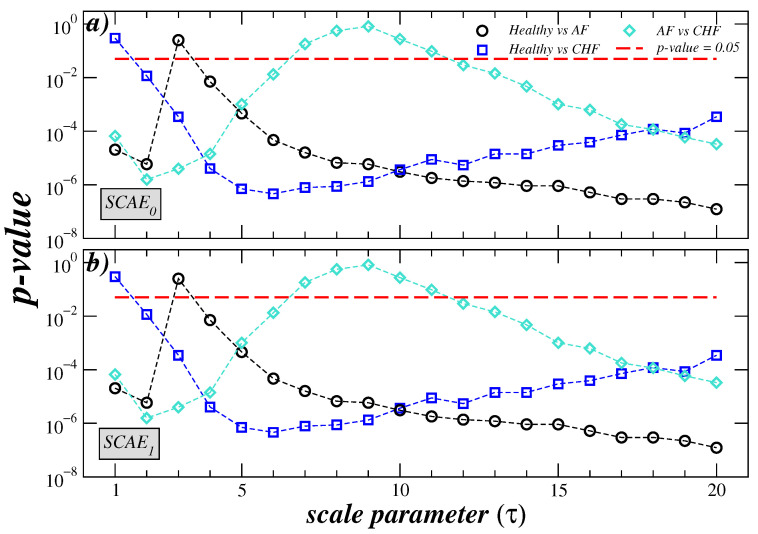
Results of *p*-values from the Mann–Whitney U *t*-test applied to the entropy values in Figure 2. The healthy vs. AF, healthy vs. CHF, and AF vs. CHF cases for <SCAE_0_> (**a**) and <SCAE_1_> (**b**) are shown. The dashed red line represents the critical value p=0.05.

In order to assess whether the distributions of the three pairs of SCAE value groups are differentiated across the entire range of scales, the non-parametric Mann–Whitney U test was used (similar results of significant difference between the three pairs of groups were found using the Student’s test, however, the normality test is not satisfied for the entire range of scale factors). The behavior of the *p*-value for the three possible comparisons between the groups is shown in Figure 3. For all scales, the SCAE_0_ and SCAE_1_ values of the healthy subjects are significantly higher (p<0.05) than those of the CHF subjects, except for τ=1. Additionally, the SCAE_0_ and SCAE_1_ values of healthy subjects were found to be significantly lower than those of AF patients for scales 1 and 2, while they were significantly higher for scales greater than or equal to 4. Furthermore, the SCAE_0_ and SCAE_1_ values of CHF patients were found to be significantly lower than those of AF patients for scales less than 6, while SCAE_0_ (SCAE_1_) values were significantly higher for scales greater than 13.

**Figure 4:**
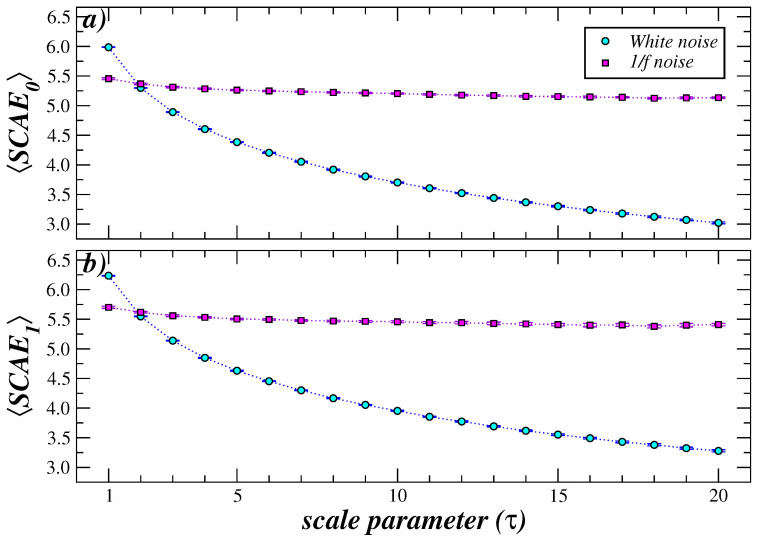
MS-SCAE analysis of correlated (1/f noise) and white noise time series. The average SCAE was computed over 10 independent realizations at each scale τ. The symbols represent the mean value for 10 independent realizations and the error bars represent the standard error ($SD/\sqrt{n}$, with *n* the number of realizations). In both cases SCAE_0_ (**a**) and SCAE_1_ (**b**), for the original series (when τ=1), the white noise has a higher associated entropy value than the correlated noise. For τ≥2, the former decreases monotonically while the latter remains approximately constant.

Next, we test the MS-SCAE on correlated 1/f and uncorrelated white noise. The results are displayed in Figure 4. Our results show that while the entropy remains relatively constant for correlated noise, the entropy decreases rapidly for uncorrelated noise, confirming that the MS-SCAE analysis is able to capture the level of complexity across multiple scales. In addition, we observe that the entropy decay associated with white noise is much faster in the MS-SCAE compared to the typical MSE analysis, so that the SCAE crosses the white noise value at scale τ=2, while for MSE it occurs at τ=4 (see Figure 3 of ref. [6]). This behavior is attributed to the fact that the coarse-graining procedure, in combination with the construction of the simplicial complex required in the SCAE calculation, exhibits greater sensitivity compared to that observed in the conventional MSE analysis.

**Figure 5:**
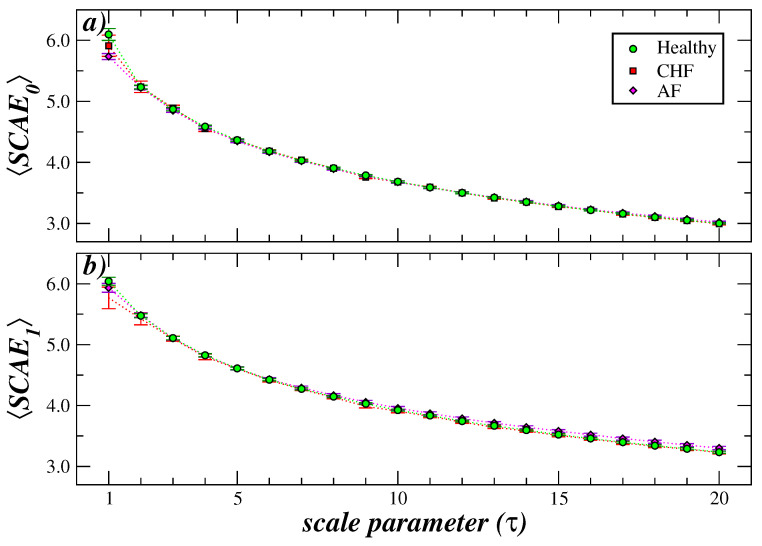
MS-SCAE Analysis of randomized versions of heartbeat time series. It is observed that the behavior of the entropies, either SCAE_0_ (**a**) or SCAE_1_ (**b**), resembles the behavior of white noise.

We also tested the MS-SCAE on the randomized versions of the heartbeat sequences from the three groups, where temporal correlations have been destroyed (see Figure 5). We find that both entropies, SCAE_0_ and SCAE_1_, have higher values for small scales, but experience a rapid decay as the scale increases, indicating a loss of complexity with a profile similar to that of white noise. The collapse of the three analyzed groups into a single behavior confirms that the correlations present in the original sequences have been destroyed.

## 4. Comparison with Other Multiscale Methods and Length of the Time Series

**Figure 6:**
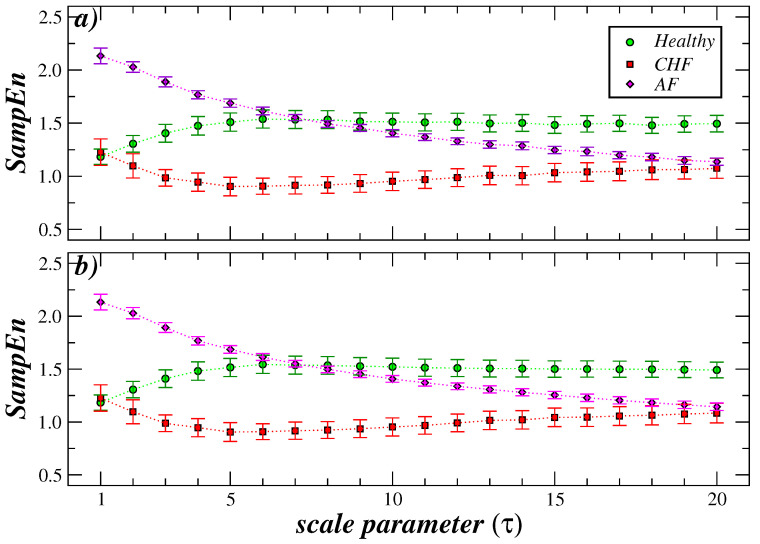
Multiscale entropy analysis (MSE) and modified multiscale entropy analysis (MMSE) of heartbeat time series. (**a**) MSE analysis of healthy subjects, patients with congestive heart failure (CHF) and patients with atrial fibrillation (AF). (**b**) As in (**a**) but using MMSE. The symbols represent the mean value, and the error bars represent the standard error.

In order to contextualize the results of the MS-SCAE analysis, we compare them to the results of multiscale entropy (MSE) and modified multiscale entropy (MMSE) [37,43]. To have a direct comparison, we reproduced these calculations for the data set analyzed in this paper. These results are displayed in Figure 6. As expected, MSE (Figure 6a) and MMSE (Figure 6b) have very similar behavior for the SampEn as τ increases. However, compared with the MS-SCAE, we observe particular differences. As illustrated in Figure 2, the AF data demonstrate a persistent decline, intersecting with the data from healthy subjects and CHF patients on smaller scales compared to the findings observed in the MSE and MMSE analyses. Under MS-SCAE, the AF-Healthy crossover occurs on the scale of τ=3, and the overlap includes only two scale values, τ=3 and τ=4. Conversely, under MSE, the intersection occurs on the scale of τ=7, and the overlap is maintained for eight scales (from τ=5 to τ=12). In the case of patients with AF and CHF, MS-SCAE values overlap across τ=8 to τ=17, while the overlap of the MSE and MMSE segments commences at τ=17. These results suggest that the MS-SCAE has the capacity to detect early, similar (or dissimilar) dynamics in the time series arising at the specified scales. This represents a distinct advantage over the MSE and MMSE strategies. A more quantitative comparison between MS-SCAE and MSE results for the cases analyzed in this paper can be found online in the supplementary material at https://doi.org/10.6084/m9.figshare.28856489, last accessed day: 22 April 2025.

**Figure 7:**
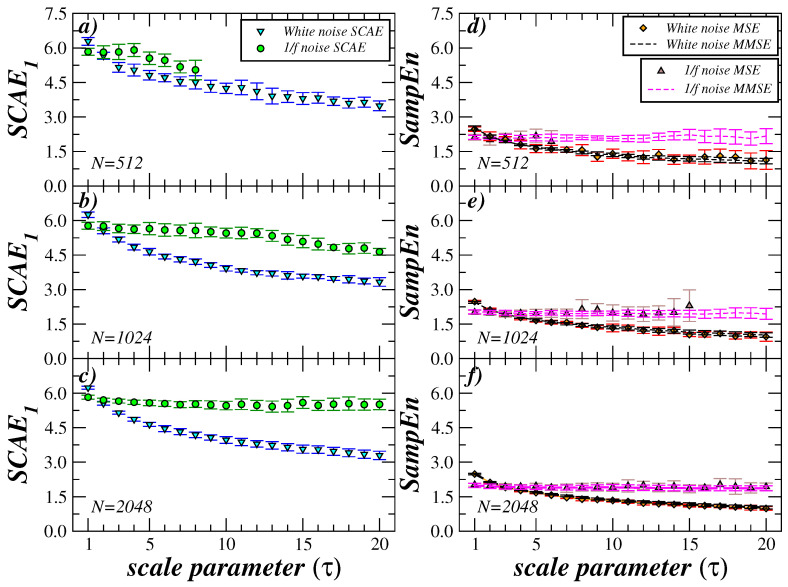
MS-SCAE_1_, MSE and MMSE for time series with different length. (**a**–**c**) MS-SCAE_1_ analysis of white and 1/f noises. (**d**–**f**) MSE and MMSE of white and 1/f noises. The symbols represent the mean value, and the error bars represent the standard error.

Finally, we investigated the dependence of the MS-SCAE analysis on the length of the time series. The SCAE_1_ results vs. τ are presented in Figure 7 for white noise and 1/f using sequences of 512 [(a) and (d)], 1024 [(b) and (e)], and 2048 [(c) and (e)] data points. Additionally, MSE and MMSE analyses were conducted for the same data sequences. As illustrated in Figure 7a, for correlated noise, SCAE1 can be estimated up to a maximum scale of 8 for MS-SCAE, while for MSE, the maximum scale achieved is 6 for a time series length of N=512. For a length of 1024, MS-SCAE provides information at the maximum scale that was analyzed τ=20 (Figure 7b). Meanwhile, MSE achieves a maximum scale of 15. When a sequence length of 2048 is employed, both MS-SCAE and MSE provide information up to the maximum scale that was analyzed (Figure 7c).

Conversely, the panels (d–f) of Figure 7 illustrate that the MMSE offers entropy information for all scales examined and all three cases of series lengths. This finding suggests a potential advantage over the conventional MSE and even over the MS-SCAE. However, it is important to note that the reliability of this result is questionable at large scales. This is due to the fact that the MMSE consists of a smoothed moving average type time series, rather than a non-overlapping window as occurs in the MSE and MS-SCAE. While MS-SCAE’s limitations, such as its inability to provide information for high scales in time series with a length of N=512 records, are noteworthy, this is a generalized numerical problem in the multiscale context.

## 5. Discussion and Conclusions

In this paper, we present the MS-SCAE method for coarse-grained heartbeat time series analysis. Our approach is based on the construction of the simplicial complex and the quantification of (*k*) and (k+1)-dimensional simplexes, and how these local structures change upon coarse-graining. We have seen that MS-SCAE is able to capture different levels of complexity at different scales, showing a clear differentiation between the group with healthy heartbeat dynamics and the groups with cardiac pathologies such as CHF and atrial fibrillation. Our results are in agreement with those obtained with the MSE method, based on the calculation of SampEn or ApEn, which show a spectrum similar to that obtained with MS-SCAE, but in the latter the entropy decreases more rapidly for the case of AF (or white noise), allowing us to identify the characteristics of the fluctuations in a smaller range of scales. Also, when comparing correlated noise and white noise, the MS-SCAE is robust in effectively capturing a rapid decrease in complexity in the uncorrelated case, while the measure is stable for the long-term memory case. These characteristics represent advantages of our approach regarding the original MSE analysis.

Unlike MSE, in our procedure we do not increase the dimensionality of the pattern to determine the matches, but we increase the dimension of the simplex to determine the unpredictability measure. The latter may represent an advantage that is reflected in the stability of the complexity indices when observed over several scales. In future work, we will explore with analytical calculations the behavior of the MS-SCAE for correlated and uncorrelated noise cases, as well as its relationship with other time series complexity measures.

Furthermore, we also stress the scientific relevance of MS-SCAE, which is characterized by its ability to bridge the gap between temporal and topological analyses. This ability provides a quantification of complexity at various scales of the time series. Additionally, SCAE complements MSE or MMSE analyses. In conclusion, our study has demonstrated that multiscale analysis based on simplicial complex entropy allows us to characterize the complexity of cardiac interbeat sequences, differentiating healthy dynamics from those with pathology. Our results highlight the importance of understanding the complexity in cardiac dynamics and its changes or degradation with disease or aging. Future research directions in MS-SCAE analysis should involve extending its theoretical framework to more accurately capture complex behavior across diverse time series domains. Applications in fields such as neural dynamics, financial market behavior, and climate variability offer promising avenues for demonstrating its utility. The enhancement of computational efficiency is a crucial aspect that merits attention in future applications, as it facilitates the characterization of complex dynamical systems.

## Data Availability

The data used in this study are available in Physionet at www.physionet.org (last accessed date: 1 December 2024). Examples of MS-SCAE analysis for some representative cases can be seen in Python code in the Github repository: https://github.com/huitzo/cslupiita/tree/msscae (last accessed date: 5 April 2025).
